# Paired comparison of the analytical performance between the Oncomine™ Comprehensive Assay v3 and whole-exome sequencing of ovarian cancer tissue

**DOI:** 10.1007/s11033-024-09715-y

**Published:** 2024-07-17

**Authors:** Joanna Lopacinska-Jørgensen, Lau K. Vestergaard, Lone Schejbel, Claus K. Høgdall, Tim Svenstrup Poulsen, Estrid V. Høgdall

**Affiliations:** 1 Department of Pathology, Herlev Hospital, University of Copenhagen, Borgmester Ib Juuls Vej 25, Herlev, 2730 Denmark; 2 Department of Gynaecology, Juliane Marie Centre, Rigshospitalet, University of Copenhagen, Copenhagen, Denmark

**Keywords:** Somatic variants, Targeted gene panel, Whole-exome sequencing, Ovarian cancer tissue

## Abstract

**Background:**

Next-generation sequencing (NGS) has been implemented in clinical oncology as a personalized medicine tool to identify targetable genetic alterations and to guide treatment decisions. However, the optimal NGS test strategy and target genes for clinical use are still being discussed. The aim was to compare the performance of the Oncomine™ Comprehensive Assay v3 (OCAv3) (targeted gene panel) and whole-exome sequencing (WES) to investigate somatic single and multiple nucleotide variants and small indels in ovarian cancer patients.

**Methods and results:**

Genomic DNA was isolated from fresh frozen samples of five high-grade serous (HGSC) and three clear cell ovarian (oCCC) cancer patients. Exome sequencing libraries were prepared by using the Ion AmpliSeq Exome RDY kit, whereas libraries for OCAv3 were prepared using by Ion AmpliSeq™ Library Kit Plus. Sequencing was performed using the Ion S5XL System (Thermo Fisher Scientific). When including only variants classified as pathogenic, likely pathogenic or unknown significance based on ClinVar database verdicts and comparing overlapping regions covered both by the OCAv3 assay and WES, 23 variants were detected by both assays. However, OCAv3 detected additionally two variants: *ARID1A*: p.Gln563Ter and *TP53*: p.Ser261ValfsTer84 that have not passed WES filtering criteria due to low coverage.

**Conclusions:**

With the present treatment possibilities, OCAv3 panel testing provided higher diagnostic yield due to better coverage. Our study emphasizes that WES, although offering the potential to identify novel findings in genes not covered by OCAv3, might overlook variants in genes relevant for OC.

**Supplementary Information:**

The online version contains supplementary material available at 10.1007/s11033-024-09715-y.

## Introduction

Epithelial ovarian cancer (EOC), which constitutes 90% of ovarian cancer (OC) cases, is the leading cause of death in women with gynecologic cancer, as up to 70% is diagnosed in late, more advanced stages (Federation of Gynecology and Obstetrics (FIGO III-IV)) [1]. The 5-year survival rate depends highly on the stage at the diagnosis. Hence, early stage (FIGO I) patients display a survival rate above 90%, while for late stages (FIGO IV) it is below 30% [2]. EOC can be categorized into five distinct subtypes: high-grade serous (HGSC, 70%), clear cell (oCCC, 10%), endometrioid (10%), mucinous (< 5%), and low-grade serous carcinomas (< 5%) [3]. These subtypes display different morphological, genetic, epigenetic, and clinical features [3]. For example, HGSC, which is the most common subtype of EOC, is characterized by *TP53* mutations (96% cases) and alterations in homologous recombination (HR) DNA damage response pathway (DDR) (approximately 50% cases) [4]. Contrary to HGSCs, oCCCs usually present a lower frequency of HR gene alterations and express wild-type p53 protein [5]. Genetic alterations in *ARID1A* and *PIK3CA* as single or double-hit mutations are most frequent in oCCC patients (approximately 50%) [6].

Diverse next-generation sequencing (NGS) approaches such as whole genome sequencing (WGS), whole exome sequencing (WES), RNA sequencing or targeted gene panels are widely used in cancer research and diagnostics worldwide [7]. WGS or WES offer a potential to identify molecular biomarkers, especially to be used in new clinical trials for being validated in future standardized or experimental treatment options, whereas targeted gene panels, which provide greater depth of coverage by focusing on known cancer associated genes, are used routinely in diagnostics to guide personalized treatment [7, 8]. There is ongoing discussion which of these approaches should be implemented in a diagnostic routine use, as there are many factors to consider, e.g., time, cost, confidence of discovered variants, data-analysis effort of large datasets if not in-silico based strategies are used and low-likelihood of benefits [9, 10]. Moreover, each of these sequencing approaches consists of a chain of various biochemical steps and different data filtering and analysis strategies that may impact coverage and variant calling accuracy among vendors [11]. DNA quality and extraction methods, library preparation, the sequencing platform, coverage, data filtering and analysis workflow are among main factors that might impact final sequencing results in clinical context, where formalin-fixed paraffin-embedded tissues are routinely used [11, 12].

Recently, we have published results of NGS in OC patients based on two approaches: target gene panel (OCAv3) [13, 14] and WES [15]. Here, we present a direct comparison between OCAv3 and WES including detection of somatic single and multiple nucleotide variants (SNV and MNV), as well as small insertions/deletions (INDELs) in exonic and splice site regions of 146 OCAv3 genes in 5 HGSC and 3 oCCC patients. In order to limit the number of factors that could possibly contribute to any variation, we decided to perform the comparison of two strategies (OCAv3 and WES) by use of fresh-frozen tissues although formalin-fixed paraffin-embedded tissues are main source for tumor molecular characterization in a daily clinical routine. However, formalin treatment might lead to a range of chemical modifications to the DNA, thereby causing technical challenges and affecting the accuracy of sequencing [16]. The libraries for both strategies were prepared from the same DNA sample for each sample.

Moreover, the two strategies are offered by the same vendor, which offers a possibility to have same library preparation strategy, and sequencing platform, as well as comparable data filtering and analysis workflow. The choice of the vendor was based on the current platform being used for routine clinical testing in our department.

## Materials and methods

### Patient cohort

The fresh frozen samples of five high-grade serous (HGSC) and three clear cell ovarian (oCCC) cancer patients samples were acquired from two Danish projects: the Pelvic Mass study (2004–2014) and the GOVEC (Gynecological Ovarian Vulva Endometrial Cervix cancer) study (2015 – ongoing) through the Bio- and Genome Bank Denmark. The oCCC_02 sample was described as mixed clear-cell and endometroid histology although with dominant oCCC histology. The study was performed according to the guidelines of the Declaration of Helsinki, including written informed consent from all patients. The study has been approved by the Danish National Committee for Research Ethics, Capital Region (H-17,029,749/H-15,020,061). To determine percentage of tumour cells, a pathologist specialized in gynecology examined haematoxylin and eosin (H&E)-stained tissue slides neighboring the excised tumor.

### Wes and OCAV3 sequencing

Genomic DNA was extracted from fresh frozen samples using Maxwell RSC Tissue DNA (AS1610, Promega). DNA concentration measurements were performed on the Qubit system with the High Sensitivity dsDNA assay kit (Q33120, Thermo Fisher Scientific). Exome sequencing libraries were prepared from 100 ng DNA using the Ion AmpliSeq Exome RDY kit (A38262, Thermo Fisher Scientific) according to the manufacturer’s protocol. The Oncomine™ Comprehensive Assay v3 (OCAv3) libraries were prepared according to the manufacturer’s instructions MAN0015885 (Revision C.0) with Ion AmpliSeq™ Library Kit Plus (Thermo Fisher Scientific). Multiplex PCR amplification was conducted using a DNA concentration of approximately 20 ng as input for OCAv3 assay. Amplified exome and OCAv3 DNA libraries were loaded onto an Ion 550 Chip (A34537, Thermo Fisher Scientific) using the Ion Chef System (Thermo Fisher Scientific). Sequencing was performed on an Ion S5XL System (Thermo Fisher Scientific).

### Data processing and variant calling

Exome sequencing data were acquired, pre-processed, aligned to the human genome assembly 19 and analyzed by Ion Reporter™ Software (v. 5.10) (Thermo Fisher Scientific), coupled with AmpliSeq Exome single sample (Somatic) analysis module. For OCAv3, Ion Reporter™ Software (v. 5.18) (Thermo Fisher Scientific), coupled with Oncomine Comprehensive v3 - w4.2 - DNA - Single Sample analysis module was used for initial automated analysis. Files were downloaded without any filter chain to include all identified variants. Further filtering for true variants was performed using R environment and Python programming language (3.9.2) [17], as described previously [14, 15], albeit with modifications regarding “Potential germline” (Allele ratio on target allele ≥ 0.98 instead of Allele ratio on target allele = 1) and “Strand bias” (Phred-scaled *p*-value from a Fisher’s Exact Test > 55 instead of 60) to filter out false positive variants. Only SNV, MNV or INDEL variants located in exonic or splice-site (located within the first three nucleotides of the 5’ or 3’ end) regions of the 146 genes from the OCAv3 were selected for further analysis. Moreover, these variants had to pass the Ion Reporter™ Default Variant View filter and their nucleotide length should be equal or above 1. *TP53* variants are found in more than 90% of HGSC cases based on previous reports [18], therefore in order to determine cut-off for a coverage filter for WES, we performed first manual *TP53* variant check of non-filtered data from the subjects with HGSC (value = 49). The following thresholds define subsequently applied exclusion criteria in OCAv3 and WES workflows:

- UCSC Common SNPs (SNPs with a minor allele frequency of at least 1% and mapped to a single location in the reference genome assembly) = “CommonSNP”.
- Ion Reporter^™^ Variant Effect = “Synonymous”.
- Coverage < 100 (OCAv3) or Coverage < 49 (WES) = “Low overall coverage”.
- Coverage < 10% of mean coverage above 100 (OCAv3) or 49 (WES) = “Low base coverage”.
- Allele ratio on target allele ≥ 0.98 = “Potential germline”.
- Homopolymer length > 5 = “High homopolymer content”.
- Allele ratio < 25% of average allele ratio per sample = “Allele ratio below Q1”.
- Phred score < 200 (OCAv3) or < 100 (WES) = “Low Phred score”.
- Ion Reporter^™^*p*-value > 0.01 = “Above p-value”.
- Phred-scaled *p*-value from a Fisher’s Exact Test > 55 = “Strand bias”.

All variants that passed the above-described criteria were clinically annotated using the ClinVar database (data status check: March 14, 2023). Variants classified as “Benign” or “Likely Benign” by the ClinVar were excluded from further analysis. All remaining variants were manually assessed for sequencing and annotation errors with integrated genomic viewer (IGV) (Broad Institute, USA) to confirm or exclude findings.

### Genomic ranges filtering

The OCAv3 enables DNA-targeted sequencing of 146 cancer-associated genes (Online Resource 1). To compare variants in genomic regions covered by both assays, we downloaded the files from the Ion Reporter™ Software (“amplicons_low_no_coverage_statistics.txt”) and extracted mutually covered locus positions for 146 genes for both OCAv3 and WES. Furthermore, a cross-check for gene names was performed and two gene names from WES panel were updated to newly approved gene names from OCAv3: *H3F3A* and *HIST1H3B* were replaced with *H3-3A* and *H3C2*, respectively.

## Results

OCAv3 sequencing was performed according to our routine protocol used to support patient diagnosis and treatment decisions where we aim for at least 8 million reads per sample which corresponds to a mean coverage of approximately 2400. For OCAv3 sequencing, the mean of mapped reads was 11.97 (± 3.40) million and mean coverage depth 3527.63 (± 1053.58) (Table 1). These numbers are in line with our previously described results of OCAv3 sequencing of 50 FFPE samples, which resulted in mean of 11.41 (± 4.44) million mapped reads and mean coverage depth of 3100.16 (± 1194.72) [14]. For WES, we aimed at 200x coverage according to the Ion AmpliSeq™ Exome RDY Library Preparation User Guide. The obtained values are not ideally in line with the expectations, as for WES there were 5 samples below expected 200x coverage (Table 1).

**Fig. 1:**
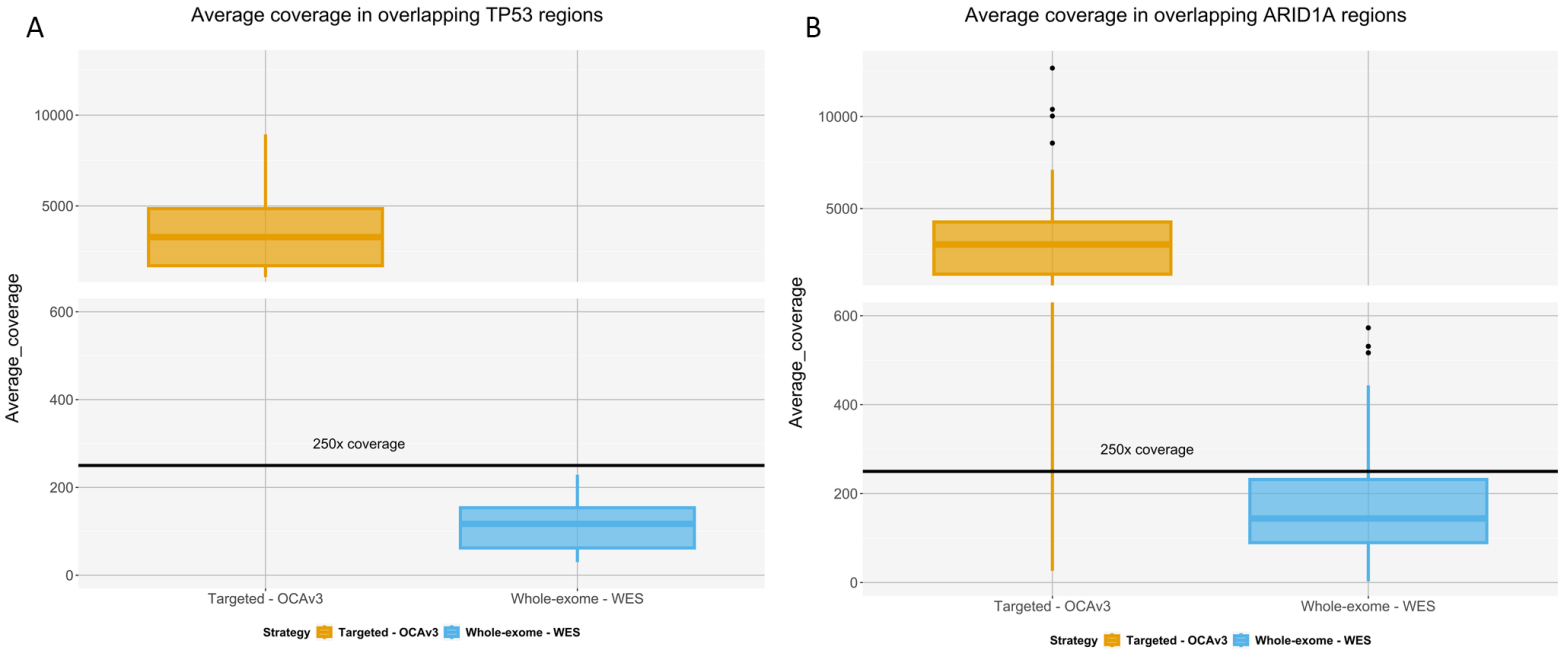
The average coverage of overlapping TP53 (A) and ARID1A (B) regions in eight samples sequenced by both assays (OCAv3 and WES). Horizontal black line indicates coverage of 250x, which is suggested as satisfactory for detection of somatic variants [19]. Note that y-axis is not continuous to display both strategies on the same plot. The coverage for each overlapping region between OVAv3 and WES was averaged across eight samples. Outliers (black dots) are defined as values exceeding 1.5 times the interquartile range above the 75th percentile or falling below 1.5 times the interquartile range below the 25th percentile

Among 25 variants detected by OCAv3 panel (pathogenic, likely pathogenic or variants of uncertain significance) (Table 2), two variants have not passed filtering criteria for WES: *ARID1A*: p.Gln563Ter and *TP53*: p.Ser261ValfsTer84 due to low coverage (Table 3). Both variants are not present in the ClinVar database. The mean coverage for the OCAv3 assay is 1707, whereas for WES is 189, when considering all targeted regions specific for each assay. However, when comparing the mean coverage per gene, there are some differences, for example the average coverage for targeted regions of *TP53* is 120, whereas for *ARID1A* is 181 for WES (Fig. 1 and Online Resource 2). Not all regions of both genes are covered uniformly for both assays (Fig. 1 and Online Resource 2). All *TP53* and 2 out of 99 *ARID1A* OCAv3 amplicons have coverage above 100x. Conversely, 2 out of 14 *TP53* and 7 out of 43 *ARID1A* WES amplicons had coverage below 49 (Online Resource 2).

There is a significant difference of coverage for these two variants for both assays (1994 versus 25 for the *ARID1A* variant, and 1756 versus 37 for the *TP53* variant) (Table 3). Both variants are associated with high frequency > 83%. The tumor content estimated by the pathologist for oCCC_01 was 40%, and 50% for oCCC_03. There was another sample with similar tumor content: HGSC_05 – 40% and no differences between the variants were reported for the two strategies: OCAv3 and WES (Table 1).

There were no variants that were reported only by WES and not by OCAv3 in the overlapping regions of the 146 shared genes.

**Table 1:** Sequencing metrics for Oncomine™ Comprehensive Assay v3 and whole-exome sequencing

Sample ID	Tumor percentage	Total number of Reads	Mean Coverage Depth (fold)	Total number of Reads	Mean Coverage Depth (fold)
		Oncomine™ Comprehensive Assay v3		Whole-exome sequencing	
**HGSC_01**	70	7,360,052	2121	65,858,112	202
**HGSC_02**	80	16,286,800	4836	57,411,806	174
**HGSC_03**	65	8,809,690	2633	70,032,732	212
**HGSC_04**	80	16,445,914	4861	71,870,582	216
**HGSC_05**	40	8,324,914	2259	57,874,875	176
**oCCC_01**	40	10,867,350	3227	48,791,876	153
**oCCC_02**	70	12,954,078	3862	54,808,402	164
**oCCC_03**	50	14,693,872	4422	63,544,143	169

**Tumor percentage**: The tumor content estimated by the pathologist; **Total Number of Reads**: The total number of reads; **Mean Coverage Depth (fold)**: The mean depth of coverage

**Table 2:** Summary of pathogenic (P), likely-pathogenic (LP), or variants of unknown significance (VUS) found by whole-exome (WES) and/or Oncomine™ Comprehensive Assay v3 (OCAv3) sequencing in exonic and splice-site regions of 146 OCAv3 genes that were covered by both assays

Sample ID	Genes	Exon	Locus	Transcript (WES)	Transcript (OCAv3)	Coding	Amino Acid Change	ClinVar	Variant Effect	Found by:
**oCCC_01**	***ARID1A***	**3**	**chr1:27057979**	**NM_006015.5**	**NM_006015.6**	**c.1687 C > T**	**p** **.Gln563Ter**	**ND**	**nonsense**	**OCAv3**
**oCCC_03**	***TP53***	**7**	**chr17:7577500**	**NM_000546.5**	**NM_000546.6**	**c.780delC**	**p** **.Ser261ValfsTer84**	**ND**	**frameshiftDeletion**	**OCAv3**
HGSC_01	*BRCA1*	10	chr17:41244690	NM_007300.3	NM_007294.4	c.2857delT	p.Cys953ValfsTer47	P	frameshiftDeletion	OCAv3&WES
HGSC_01	*KIT*	18	chr4:55602680	NM_000222.2	NM_000222.3	c.2501 A > G	p.Lys834Arg	ND	missense	OCAv3&WES
HGSC_01	*TP53*	5	chr17:7578454	NM_000546.5	NM_000546.6	c.476 C > T	p.Ala159Val	P/LP	missense	OCAv3&WES
HGSC_02	*FANCD2*	27	chr3:10114647	NM_033084.4	NM_033084.6	c.2587 A > G	p.Arg863Gly	ND	missense	OCAv3&WES
HGSC_02	*IGF1R*	17	chr15:99478545	NM_000875.4	NM_000875.5	c.3187G > T	p.Val1063Leu	ND	missense	OCAv3&WES
HGSC_02	*TP53*	6	chr17:7578203	NM_000546.5	NM_000546.6	c.646G > A	p.Val216Met	CIP (P:4 LP:14 VUS:2)	missense	OCAv3&WES
HGSC_03	*BRCA1*	10	chr17:41246748	NM_007300.3	NM_007294.4	c.799delT	p.Ser267GlnfsTer31	P	frameshiftDeletion	OCAv3&WES
HGSC_03	*FBXW7*	3	chr4:153271242	NM_033632.3	NM_033632.3	c.536G > A	p.Arg179His	ND	missense	OCAv3&WES
HGSC_03	*TP53*	6	chr17:7578190	NM_000546.5	NM_000546.6	c.659 A > C	p.Tyr220Ser	P	missense	OCAv3&WES
HGSC_04	*CDK12*	14	chr17:37687396	NM_016507.3	NM_016507.4	c.4300_4301delGAinsTT	p.Glu1434Leu	ND	missense	OCAv3&WES
HGSC_04	*TP53*	4	chr17:7579591	NM_000546.5	NM_000546.6	c.97-1G > A	p.?	LP	unknown	OCAv3&WES
HGSC_05	*FANCA*	42	chr16:89805301	NM_000135.3	NM_000135.4	c.4249 C > G	p.His1417Asp	CIP (P:1 VUS:2 LB:4 B:7)	missense	OCAv3&WES
HGSC_05	*MRE11*	13	chr11:94192599	NM_005591.3	NM_005591.4	c.1475 C > A	p.Ala492Asp	CIP (VUS:3 LB:9 B:6)	missense	OCAv3&WES
HGSC_05	*TP53*	5	chr17:7578517	NM_000546.5	NM_000546.6	c.413 C > T	p.Ala138Val	CIP (LP:1 VUS:1 )	missense	OCAv3&WES
oCCC_01	*PIK3CA*	21	chr3:178952085	NM_006218.3	NM_006218.4	c.3140 A > G	p.His1047Arg	P	missense	OCAv3&WES
oCCC_01	*RB1*	3	chr13:48916811	NM_000321.2	NM_000321.3	c.341 C > T	p.Ser114Leu	CIP (VUS:1 B:1)	missense	OCAv3&WES
oCCC_01	*TSC2*	42	chr16:2138465	NM_000548.4	NM_000548.5	c.5278T > C	p.Tyr1760His	VUS	missense	OCAv3&WES
oCCC_02	*ARID1A*	3	chr1:27058069	NM_006015.5	NM_006015.6	c.1780delC	p.Gln594SerfsTer25	ND	frameshiftDeletion	OCAv3&WES
oCCC_02	*AXL*	3	chr19:41727077	NM_021913.4	NM_021913.5	c.335 C > T	p.Thr112Met	VUS	missense	OCAv3&WES
oCCC_02	*H3C2*	1	chr6:26032230	NM_003537.3	NM_003537.4	c.59 A > T	p.Gln20Leu	ND	missense	OCAv3&WES
oCCC_02	*PIK3CA*	2	chr3:178916956	NM_006218.3	NM_006218.4	c.344_346 delGAG	p.Arg115_Glu116 delinsGln	ND	nonframeshiftDeletion	OCAv3&WES
oCCC_02	*PTEN*	5	chr10:89692904	NM_000314.6	NM_000314.8	c.388 C > G	p.Arg130Gly	P	missense	OCAv3&WES
oCCC_02	*TP53*	7	chr17:7577550	NM_000546.5	NM_000546.6	c.731G > A	p.Gly244Asp	P	missense	OCAv3&WES

^1^ND – no data available for a variant in ClinVar, ^2^CIP – Conflicting interpretations of pathogenicity in ClinVar

**Table 3:** Comparison of the coverage and allele frequency for variants that were found by the Oncomine™ Comprehensive Assay v3 (OCAv3) workflow, but have not passed minimum coverage filtering criteria for whole-exome sequencing (WES)

						OCAv3			WES		
Sample_ID	Genes	Locus	Ref	Observed Allele	Type	Allele Frequency [%]	Coverage	Allele Coverage	Allele Frequency [%]	Coverage	Allele Coverage
oCCC_01	***ARID1A***	chr1:27057979	C	T	SNV	87.86	1994	C = 242, T = 1752	96.00	25	C = 1, T = 24
oCCC_03	***TP53***	chr17:7577500	TG	T	INDEL	87.7	1756	TG = 216, T = 1540	83.78	37	TG = 6, T = 31

## Discussion

Targeted gene panels such as the OCAv3 panel, which covers 146 cancer-associated genes, display many advantages such as lower cost, short turnaround time, low rate of unspecific or incidental findings, and a high depth of coverage as well as using formalin fixed and paraffin embedded tissue from routine pathology setting. However, they may be less applicable for discovery studies than WES or WGS approaches [20]. The generation of large datasets requires additional computational power, data analysis if all data are analysed and also storage costs. Special consideration for research studies has to be paid to novel or secondary findings in regards to their potential impact [21].

Although there is a lot of discussion regarding the benefits and the disadvantages of both investigated approaches, there are limited reports, which compare the diagnostic performance of targeted gene panels versus WES [20]. Therefore, we compared the performance of OCAv3 and WES, while detecting somatic SNVs, MNVs, and INDELs in exonic and splice-site regions of all 146 OCAv3 genes in five HGSC and three oCCC patients. Our study indicates there is a risk of missing variants in clinically relevant genes when performing the WES testing, for example when comparing the coverage differences between two assays for the *TP53* gene (Fig. 1A) or the *ARID1A* gene (Fig. 1B). Indeed, when comparing with the OCAv3 assay, two variants were not found by WES: *ARID1A*: p.Gln563Ter and *TP53*:p.Ser261ValfsTer84 (Table 3). Variant classification is not a straightforward task as until now there is no gold standard method for determining variant pathogenicity. Consequently, various resources gathering population data, functional information, disease databases and scientific reports are used by scientists and clinicians to categorize variants [22]. The ClinVar database (http://www.ncbi.nlm.nih.gov/clinvar/) at the National Center for Biotechnology Information is a freely available archive of submitted interpretations of the clinical significance of variants [23]. Interestingly, interpretations for a same variant might disagree, as shown in Table 2 for some variants: e.g., *TP53*:p.Val216Met or *FANCA*:p.His1417Asp, which emphasizes the need for comprehensive set of standards for variant classification [24]. Both missing variants: *ARID1A*:p.Gln563Ter and *TP53*:p.Ser261ValfsTer84 were not present in the ClinVar database, but they would be classified in clinical routine practice as likely oncogenic/oncogenic based on the “Standards for the classification of pathogenicity of somatic variants in cancer” recently published as joint recommendations of Clinical Genome Resource (ClinGen), Cancer Genomics Consortium (CGC), and Variant Interpretation for Cancer Consortium (VICC) [25]. The *TP53*:p.Ser261ValfsTer84 variant was recently reported as likely oncogenic in Brazilian patients EOC cohort [26].

The missing variants are not associated with low allele frequencies (Table 3). In case of OC, variants are subtype-specific, therefore based on the sequencing analysis it is possible to confirm or exclude an initial diagnosis. HGSC is characterized by *TP53* mutations (96% cases), whereas variants in *ARID1A* and *PIK3CA* are most frequent in oCCC patients (approximately 50%) [5, 6]. Satisfactory coverage (preferably 250) for detection of somatic variants for WES sequencing is associated with higher cost regarding consumables, turnaround time, data analysis and storage [19]. Moreover, increasing the overall coverage for WES sequencing might not be sufficient enough, as the reduced coverage in very specific regions will remain the problem for precise variant calling [27, 28]. As presented in the study, the expected and achieved coverage were different for more than 50% of samples, therefore the real values need to be determined empirically in specific clinical settings. The overall coverage for *TP53* gene in overlapping regions is 120 for WES (Online Resource 2), but for particular regions it might be below the coverage threshold, which might lead to omitting potentially relevant variants, as shown in our study. However, this appears to only impact a subset of variants, as other variants from *TP53*:p.Gly244Asp located on exon 7 and *ARID1A*:p.Gln594SerfsTer25 located on exon 3 were detected using WES showing the highly variable coverage across same exons. Therefore, before implementing any of sequencing strategies in a clinical context, evaluation of the potential influence of reduced coverage on the clinically relevant regions of the genome needs to be performed.

In order to perform targeted NGS sequencing, target enrichment is required and it can be accomplished by using two major approaches: PCR-based amplicon and hybrid capture-based methods [19]. PCR amplification has been highly effective in sequencing applications where the nucleic acids are scarce or of poor quality, such as fine needle aspirates or formalin-fixed paraffin embedded tissues. Moreover, it offers short, simple and cost-effective workflow. However, when compared to hybrid-capture-based methods, PCR-based amplicon method might result in higher rate of sequencing errors, for example in regions rich in repetitive sequences [19, 29, 30].

Our study has been focused on the analytical performance between the OCAv3 and WES and have been performed on the same isolated DNA per each sample, based on same target enrichment strategy (PCR-based amplicon) and were sequenced by use of the same platform. Therefore, it would be beneficial to compare the impact of various enrichment strategies and sequencers on the final list of detected variants, while working with OC, as such impact has been reported previously, however not in OC [29].

## Conclusions

WES offers the potential to investigate nearly all protein coding regions of the genome. Therefore, it enables finding of variants in novel disease associated genes variants that might be particularly interesting for discovery of potential new treatment targets and options. Such findings may not be beneficial for individual patients if there are no approved treatment strategies available at the sequencing testing time. However, novel findings might facilitate recruitment into clinical trials if genes are not currently used to guide diagnostics or treatment decisions. When moving from exploratory WES to diagnostic WES is considered, the need for a much higher sequencing depth should to be taken into account. Therefore, to minimize costs associated with performing assay in routine diagnostic setting, data analysis and storage, targeted gene panels such as OCAv3 seem to be more suitable in clinical testing. Moreover, as we demonstrate that the differences between WES and OCAv3 in clinically relevant genes for subtype classification can be observed, each testing center needs to take into consideration the advantages and benefits of implementing these strategies for performing clinical testing [19, 31, 32].

## Electronic supplementary material

Below is the link to the electronic supplementary material.

Supplementary Material 1

Supplementary Material 2

## Data Availability

The datasets generated during and/or analysed during the current study are not publicly available, which is in accordance with the rules concerning processing of personal data set out in the EU General Data Protection Regulation (GDPR) and the Danish Data Protection Act. Nonetheless, may a researcher have an interest in our data they are welcome to contact us and collaborate. The data that support the findings of this study can be requested from The National Secretariat for Bio- and Genome Bank Denmark, RBGB.sekretariat.herlev-og-gentofte-hospital@regionh.dk, Herlev Hospital, Borgmester Ib Juuls Vej 73, 2730 Herlev, Denmark.
