# Supplementary material for: Paired comparison of the analytical performance between the Oncomine™ Comprehensive Assay v3 and whole-exome sequencing of ovarian cancer tissue
Source: Mol Biol Rep. 2024 Jul 17;51(1):820. doi: 10.1007/s11033-024-09715-y (PMC11255052; doi:10.1007/s11033-024-09715-y)
Supplement: Supplementary file 2 — Supplementary Material 2 [file 11033_2024_9715_MOESM2_ESM.docx]

**Supporting information**

***Online Resource 1 A list of 146 genes from the Oncomine™ Comprehensive Assay v3***

| *AKT1* | *CCNE1* | *EZH2* | *HRAS* | *MET* | *NTRK3* | *RAF1* | *TOP1* |
| --- | --- | --- | --- | --- | --- | --- | --- |
| *AKT2* | *CDK12* | *FANCA* | *IDH1* | *MLH1* | *PALB2* | *RB1* | *TP53* |
| *AKT3* | *CDK2* | *FANCD2* | *IDH2* | *MRE11* | *PDGFRA* | *RET* | *TSC1* |
| *ALK* | *CDK4* | *FANCI* | *IGF1R* | *MSH2* | *PDGFRB* | *RHEB* | *TSC2* |
| *AR* | *CDK6* | *FBXW7* | *JAK1* | *MSH6* | *PIK3CA* | *RHOA* | *U2AF1* |
| *ARAF* | *CDKN1B* | *FGF19* | *JAK2* | *MTOR* | *PIK3CB* | *RICTOR* | *XPO1* |
| *ARID1A* | *CDKN2A* | *FGF3* | *JAK3* | *MYC* | *PIK3R1* | *RNF43* |  |
| *ATM* | *CDKN2B* | *FGFR1* | *KDR* | *MYCL* | *PMS2* | *ROS1* |  |
| *ATR* | *CHEK1* | *FGFR2* | *KIT* | *MYCN* | *POLE* | *SETD2* |  |
| *ATRX* | *CHEK2* | *FGFR3* | *KNSTRN* | *MYD88* | *PPARG* | *SF3B1* |  |
| *AXL* | *CREBBP* | *FGFR4* | *KRAS* | *NBN* | *PPP2R1A* | *SLX4* |  |
| *BAP1* | *CSF1R* | *FLT3* | *MAGOH* | *NF1* | *PTCH1* | *SMAD4* |  |
| *BRAF* | *CTNNB1* | *FOXL2* | *MAP2K1* | *NF2* | *PTEN* | *SMARCA4* |  |
| *BRCA1* | *DDR2* | *GATA2* | *MAP2K2* | *NFE2L2* | *PTPN11* | *SMARCB1* |  |
| *BRCA2* | *EGFR* | *GNA11* | *MAP2K4* | *NOTCH1* | *RAC1* | *SMO* |  |
| *BTK* | *ERBB2* | *GNAQ* | *MAPK1* | *NOTCH2* | *RAD50* | *SPOP* |  |
| *CBL* | *ERBB3* | *GNAS* | *MAX* | *NOTCH3* | *RAD51* | *SRC* |  |
| *CCND1* | *ERBB4* | *H3-3A* | *MDM2* | *NRAS* | *RAD51B* | *STAT3* |  |
| *CCND2* | *ERCC2* | *H3C2* | *MDM4* | *NTRK1* | *RAD51C* | *STK11* |  |
| *CCND3* | *ESR1* | *HNF1A* | *MED12* | *NTRK2* | *RAD51D* | *TERT* |  |

***Online Resource 2 Average coverage of overlapping regions for TP53 and ARID1A sequenced by both OCAv3 and WES in eight patient sample***
